# Molecular docking analysis of penta galloyl glucose with the bcl-2 family of anti-apoptotic targets

**DOI:** 10.6026/97320630017861

**Published:** 2021-10-31

**Authors:** Karthick Dharmalingam, Velvizhi Dharmalingam, Satheesh Durairaj, Praveen Sharma, Selvaraj Jayaraman, Sarita Choudhary

**Affiliations:** 1 Department of Biochemistry, All India Institute of Medical Sciences, Jodhpur, Rajasthan- 342005, India; 2 Department of Chemistry, Captain Srinivasa Murthy Central Ayurveda Research institute, CCRAS, Ministry of AYUSH, Government of India, Chennai - 600106;; 3 Department of chemistry, Siddha Central Research institute, Arumbakkam, Chennai - 600106; 4 Department of Biochemistry, Saveetha Dental College and Hospitals, Saveetha Institute of Medical and Technical Sciences, Saveetha University, Chennai-600 077, India

**Keywords:** Breast cancer, apoptosis, penta galloyl glucose, molecular docking

## Abstract

Apoptosis requires cellular proteins from the B-cell lymphoma 2 (BCL-2) family linked to breast cancer. Therefore, it is of interest to document the Molecular docking analysis data of penta galloyl glucose with the bcl-2 family of anti-apoptotic targets (Bcl-2, BCL-XL, Caspase 3, and Caspase 9). Data shows that Pentagalloyl glucose have optimal binding features with Bcl-2, BCL-XL, Caspase 3, and Caspase 9 proteins with binding energy of -8.6,-7,-7.5 and 4.4 kcal/mol respectively for further consideration in this context.

## Background:

Breast cancer is the most frequent cancer among women around the world. Approximately 30% of women diagnosed with early-stage breast cancer go on to develop metastatic breast cancer, which necessitates therapy with anti-breast cancer therapeutic drugs. Although many modern anti-breast cancer medicines can slow tumor development, the effect is usually short-lived, and patients frequently acquire resistance to the anticancer drugs utilized, resulting in about half of all treated patients relapsing [1]. This fact showed that new medicines that are more effective, safe, and have the ability to expand the lifespan of breast cancer patients are required. In multi cellular organisms, the entire process of cell division and cell death is strictly regulated. When either of these biological processes is disrupted, it has an impact on normal development and homeostasis, which can lead to cancer. Cell proliferation pathways are enhanced during cancer progression, while programmed cell death or apoptotic pathways are decreased; thus, cancer has a repertoire of these failing processes [2]. Apoptosis has been researched in a variety of processes, including normal cell turnover, embryonic development, morphogenesis, aging, cell population regulation, and tumor progression, since its commencement [3]. Although apoptosis is a frequent process for removing undesired, damaged, or hazardous cells, other mechanisms such as anoikis, necroptosis, entosis, netosis, pyroptosis, or ferroptosis are also involved in programmed cell death [4]. Extrinsic stimuli such as viral infections, xenobiotics, and pollutants can trigger apoptosis, as can intrinsic factors such as DNA damage, reactive oxygen species (ROS), and reactive nitrogen species (RNS) [5]. Apoptosis is also involved in the clearance of immune cells that have been damaged by illness or noxious substances during immunological responses [6,7]. The B-cell lymphoma-2 (Bcl-2) family, which includes both pro- and anti-apoptotic proteins, is important in the mitochondrial pathway of apoptosis because it promotes the release of cytochrome c and Smac (Second mitochondrial-derived activator of caspases) into the cytosol, resulting in caspase-dependent cell death [8,9]. Bak and Bax, multi-domain proteins, and Bim, Bid, Puma, and Noxa, BH3 domain only proteins, are members of the Bcl-2 family of pro-apoptotic proteins [10]. Bcl-xl, Bcl-2, and Mcl-1 are the most well-known anti-apoptotic proteins. The BH3-only proteins induce apoptosis via interacting with Bak and Bax or binding to anti-apoptotic proteins, causing Bax and Bak to be released [11]. As a result, the ratio of anti- and pro-apoptotic proteins in the Bcl-2 family determines whether a cell lives or dies. Anti-apoptotic Bcl-2 proteins are regularly expressed in most tumor tissues and cancer cell lines [12]. Prostate cancer, breast cancer, B-cell lymphomas, and colorectal cancer, for example, all have high levels of Bcl-2. Overexpression of Bcl-2 family anti-apoptotic proteins promotes cell proliferation and survival, as well as therapeutic resistance due to apoptosis evasion. As a result, they are well-established pharmacological targets for cancer therapy. The development of anti-apoptotic protein Bcl-2 inhibitors has a lot of clinical potential. Phytochemicals, which are naturally occurring substances found in plants, are important resources for developing new medications and can also be used to treat cancer. Hence in the present study the anticancer activity of Pentagalloylglucose was identified by using insilico molecular docking analysis.

## Materials & Methods:

### Protein Preparation:

Three dimensional structure of BCL-2 (PDBid: 4MAN)BCL-XL: (PDB id:3ZK6), Caspase 3: (PDB id: 3GJQ), Caspase 9: (PDB id: 1NW9) were retrieved from PDB database. AutoDock Tools were used to prepare the protein structure. Water molecules and all non-standard residues have been eliminated from the basic structure. After that, all missing hydrogens and kollman charges were added to the system, and the finished protein receptor was saved as a pdbqt file and inserted immediately into PyRx's workspace folders [13].

### Preparation of ligand

The Pentagalloylglucose structure was downloaded from Pubchem databases in 2d SDF format. By using online smiles translator all the compounds were translated to 3d PDB format. The structures of the compounds were then loaded into PyRx and made into a ligand pdbqt file by clicking Load Molecule and create as ligand pdbqt file [14].

### Molecular docking:

AutoDock in PyRx PyRx( 0.8 ) GUI was used to check the binding capability of the selected antibiotics with each selected target. Docking provides valuable insight into the interaction between the proteins of with ligand. The structures of the Pentagalloylglucose were prepared before docking experiment by using make ligand command present in PyRx. Make macromolecule option was used to prepare the protein structures. AutoDock program along with the Lamarckian genetic algorithms (LGA) was used to perform docking experiments. The Lamarckian GA parameters used in the analysis consist of 30 independent runs, 150 size of population, a 25,000,000 energy evaluations, 27,000 number of generation, mutation rate of 0.02, and 0.8 crossover rate. Docking was carried out with in the pre-defined grid size for protein. For each ligand protein complex, individual docking procedures have been performed. The results were ranked in the order of increasing docking energies. The lowest binding energy of each cluster was assumed to be representative [15].

## Results and Discussion:

Docking experiments on Pentagalloylglucose compounds and anti-apoptotic proteins were conducted. The interactions between anti-apoptotic proteins and Pentagalloylglucose, as well as the binding energies of the complexes, were used to examine the AutoDock results. The accuracy of the AutoDock data was confirmed by looking at the anti-apoptotic proteins' lowest binding free energy and hydrogen bonds with penta galloyl glucose. Table 1(see PDF) shows the results of the docking analysis.

## Docking with BCL-2:

Bcl-2 was first discovered as an oncogene implicated in human B cell follicular lymphoma [16]. Vaux et al. found this protein's anti-apoptotic action in a system where apoptosis was caused by IL-3 deficiency in 1988 [17]. Bcl-2 was soon found to prevent apoptosis generated by a variety of stressors, including serum deprivation, heat shock, and chemotherapeutic chemicals, implying that Bcl-2 can prevent apoptosis through a common mechanism. Certain types of necrotic cell death are also inhibited by Bcl-2. Bcl-2 was docked with Pentagalloylglucose in this study [18]. The binding energies with Pentagalloylglucose are used to rate the docking results. The docking simulation of BCL-2 with Pentagalloylglucose produced six hydrogen bonds with a binding energy of 8.6 kcal/mol. Table 1 and Figure 1(see PDF) show the binding free energy (kcal/mol) for each Pentagalloylglucose interaction as well as the residues involved.

## Docking with BCL-XL:

JNK signaling targets Bcl-xL, a major antiapoptotic molecule. In response to microtubule inhibitors and other apoptotic stimuli like as ionizing radiation or chemotherapy, Bcl-xL is phosphorylated [19]. BCL-XL was chosen as one of the probable targets for molecular docking with Pentagalloylglucose in this investigation. According to the docking data, BCL-XL has a binding affinity for Pentagalloylglucose by forming three hydrogen bonds with binding energies of 7kcal/mol. It shows strong binding with bcl-xl, and the interaction of this docking is depicted in Figure 1 (see PDF).

## Docking with Caspase-3:

Caspases play an important role in programmed cell death (apoptosis). Caspase-3 is a commonly activated death protease that catalyzes the selective cleavage of a variety of important cellular proteins. Figure 1 and Table 1(see PDF) demonstrate the molecular docking results of caspase 3 with Pentagalloylglucose. Pentagalloylglucose has a high affinity for caspase 3, according to the docking data. The docking results revealed that in the creation of three hydrogen bonds, interacted with binding energies of -7.5 kcal/mol.

## Docking with Caspase-9:

Caspases-9, the most studied initiator caspase, is a crucial component in the intrinsic or mitochondrial pathway, which is activated by a variety of stimuli including as chemotherapies, stress agents, and radiation. Caspase-9 is hypothesized to involve homo-dimerization monomeric zymogens and is triggered on the apoptosome complex to maintain catalytic state. Failure to activate caspase-9 has far-reaching physiological and pathophysiological consequences, including degenerative and developmental problems, as well as cancer. Caspase-9 docking studies with Pentagalloylglucose revealed an interaction with a binding energy of -4.4kcal/mol and the formation of five hydrogen bonds. All these interaction were shown in the Figure 1(see PDF) and the details of interaction were shown in Table 1(see PDF). Docking simulation research revealed that all of the identified targets implicated in apoptotic proteins had a robust interaction behavior with Pentagalloylglucose in this investigation. It revealed that Pentagalloylglucose could be a promising anticancer candidate worthy of future investigation.

## Conclusion:

Our findings show that Pentagalloylglucose have optimal binding features with Bcl-2, BCL-XL, Caspase 3, and Caspase 9 proteins with binding energy of -8.6,-7,-7.5 and 4.4 kcal/mol respectively for further consideration in this context.
